# MARCH5 restores endothelial cell function against ischaemic/hypoxia injury via Akt/eNOS pathway

**DOI:** 10.1111/jcmm.16386

**Published:** 2021-02-21

**Authors:** Wenhua Lei, Junli Li, Changming Li, Li Chen, Fangyang Huang, Dan Xiao, Jialiang Zhang, Jiahao Zhao, Guoyong Li, Tianyi Qu, Hao Zhou, Yanbiao Liao, Mao Chen

**Affiliations:** ^1^ Department of Cardiology West China Hospital Sichuan University Chengdu China; ^2^ Laboratory of Cardiovascular Diseases Regenerative Medicine Research Center West China Hospital Sichuan University Chengdu China

**Keywords:** endothelial cells, endothelial nitric oxide synthase, MARCH5, myocardial infarction

## Abstract

MARCH5 is a critical regulator of mitochondrial dynamics, apoptosis and mitophagy. However, its role in cardiovascular system remains poorly understood. This study aimed to investigate the role of MARCH5 in endothelial cell (ECs) injury and the involvement of the Akt/eNOS signalling pathway in this process. Rat models of myocardial infarction (MI) and human cardiac microvascular endothelial cells (HCMECs) exposed to hypoxia (1% O_2_) were used in this study. MARCH5 expression was significantly reduced in ECs of MI hearts and ECs exposed to hypoxia. Hypoxia inhibited the proliferation, migration and tube formation of ECs, and these effects were aggravated by knockdown of MARCH5 but antagonized by overexpressed MARCH5. Overexpression of MARCH5 increased nitric oxide (NO) content, p‐eNOS and p‐Akt, while MARCH5 knockdown exerted the opposite effects. The protective effects mediated by MARCH5 overexpression on ECs could be inhibited by eNOS inhibitor L‐NAME and Akt inhibitor LY294002. In conclusion, these results indicated that MARCH5 acts as a protective factor in ischaemia/hypoxia‐induced ECs injury partially through Akt/eNOS pathway.

## 1 INTRODUCTION

Cardiovascular diseases (CVDs) are the leading cause of morbidity and mortality worldwide.^1^ The World Health Organization (WHO) documents that ischemic heart disease (IHD) is the world's biggest killer.^2^ IHD is a cardiac structural and functional abnormality caused by myocardial ischaemia and hypoxia, resulting from coronary atherosclerosis, thrombosis, vasculitis and coronary artery anomaly. Ischaemia/hypoxia injury is a common pathological basis of coronary heart disease, atherosclerosis and other CVDs. Even though various strategies have been adopted in recent decades, the need to reduce the burden of IHD is still urgent. As essential structural and functional elements of cardiovascular system, endothelial cells (ECs) regulating vascular tone, preventing platelet aggregation and inhibiting inflammatory cell adhesion, play a critical role in cardiovascular homeostasis.^3^, ^4^ Maintaining ECs function, particularly under ischaemia/hypoxia conditions, is crucial to find novel preventive and therapeutic targets for CVDs.

Reactive oxygen species (ROS) are important signalling molecules, mainly generated by mitochondria within most mammalian cells.^5^ ECs damage caused by increased levels of ROS is involved in the process of IHD and closely related to ubiquitination modification.^6^, ^7^ Ubiquitination is a ubiquitous post‐translational modification which attaches a 76‐amino‐acid peptide called ubiquitin (Ub) to substrate proteins, involved in selective protein degradation, endocytosis as well as signal transduction. The process of ubiquitination is catalysed by three types of enzymes including Ub activating enzymes (E1), Ub conjugating enzymes (E2) and Ub ligases (E3).^8^ And the selectivity of Ub system is mainly determined by E3 ligases.^9^ Many E3 ligases such as MARCH5 (membrane‐associated ring‐CH 5, also named MITOL) participated in regulation of mitochondria quality control and intracellular ROS levels.

MARCH5, located in mitochondrial out membrane (OMM), is ubiquitously expressed in various human tissues including heart, brain, liver and lung.^10^, ^11^ MARCH5 consists of four predicted transmembrane domains (TMs) and an N‐terminal C4HC3‐type RING finger domain which is critical for ubiquitin ligase activity.^10^, ^11^ Previous studies reported that MARCH5 regulate mitochondrial morphology and dynamics,^10^, ^11^, ^12^ cellular apoptosis, protein quality control, mitophagy, endoplasmic reticulum (ER) stress and protein quality‐control.^13^, ^14^, ^15^, ^16^, ^17^, ^18^, ^19^ However, whether E3 Ub ligase MARCH5 participates in ischaemia/hypoxia‐induced ECs dysfunction remains to be clarified.

Thus, the present study aimed to determine (a) the expression change of MARCH5 after myocardial infarction, (b) the potential role of MARCH5 in the pathological process of hypoxia‐induced ECs dysfunction and (c) the mechanism in the process of MARCH5‐regulated ECs function.

## 2 MATERIAL AND METHODS

### 2.1 Antibodies and reagents

Antibodies were obtained from the following commercial sources: anti‐MARCH5 (1:1000, NBP2‐21583) and anti‐MARCH5 (1:500, ab77585) were purchased from Novus and Abcam, respectively; Anti‐eNOS (1:1000, #9586), Phospho‐eNOS (Ser1177) (1:1000, #9571), Anti‐Akt (1:1000, #4691), Phospho‐Akt (Ser473) (1:1000, #4060) and Phospho‐Akt (Thr308) (1:1000, #9275) were purchased from Cell Signaling Technology; anti‐β‐actin was purchased from Proteintech; Total Nitric Oxide Assay Kit (S0023) and LY294002 (S1737) were purchased from Beyotime Biotechnology, SC79 (S7863) was obtained from Selleckchem.

### 2.2 Animal model of myocardial infarction

All animal experiments were approved in accordance with the guidelines of the Institutional Animal Care and the Ethics Committee of Sichuan University. Healthy male Sprague‐Dawley (SD) rats (200‐220 g) were randomly divided into two groups: (a) myocardial infarction (MI) model group, MI surgery was performed by ligating the left anterior descending (LAD) coronary artery with surgical sutures according to previous reports^20^; (b) sham group, in which the rats underwent a similar surgery without ligation the artery. After 7 days of ischaemia, TTC staining was used to detect myocardial infarct size as previously described.^21^

### 2.3 Echocardiography

After 7 days of the surgery, the rats were anaesthetized with isoflurane, and cardiac function was assessed by an echocardiographic imaging system (Vinno 6 Lab). The left ventricular ejection fraction (LVEF) and fractional shortening (FS) were measured.

### 2.4 Immunofluorescence and Immunohistochemistry

The immunofluorescence and immunohistochemistry procedures were performed as previously described.^22^ Briefly, hearts were excised, rinsed, fixed, embedded and then sectioned. The sections (4‐5 μm) were used. After blocked, the samples were incubated with primary antibodies overnight at 4°C. Images were captured by a light microscope and were determined via Image J (National Institutes of Health, USA), a quantitative digital analysis system. The expression of MARCH5 was evaluated by assigning percentage of positive cells and the staining intensity.

### 2.5 Cell culture and treatments

Human cardiac microvascular endothelial cells (HCMECs) were purchased from Shanghai Huzhen Biotechnology. Cells were cultured in Dulbecco's modified Eagle's medium (DMEM, Biological Industries) containing 10% foetal bovine serum (FBS, BI, Israel), penicillin (100 U/mL), streptomycin (100 U/mL) in a humidified incubator at 37°C with 5% CO_2_. Hypoxic conditions (1% O_2_, 5% CO_2_ and 94% N_2_) used fresh DMEM with 1% FBS to mimic ischemic conditions. If there's no special instruction, hypoxic cell cultures were performed for 24 hours.

### 2.6 Small interfering RNA (siRNA) transfection

Cells were seeded in the plates at 30%‐50% confluence containing medium without antibiotics before transfection. Specific small interfering RNAs (siRNA) against MARCH5 (against human) were designed and prepared by GenePharma (China), all sequences are shown in Table 1. The siRNAs were transfected into HCMECs using Lipofectamine RNAiMAX (Life Technologies) in serum‐free Opti‐MEM (Gibco). Cells transfected after 48h were harvested or used for further experiments.

**TABLE 1:** siRNA sequences of MARCH5

siRNAs	Sense (5′‐3′)	Antisense (5′‐3′)
siMARCH5‐1	GGUUUACGUCUUGGAUCUUTT	AAGAUCCAAGACGUAAACCTT
siMARCH5‐2	GCUUAGACUGUGGCGCAAATT	UUUGCGCCACAGUCUAAGCTT
siMARCH5‐3	GGGUGGAAUUGCGUUUGUUTT	AACAAACGCAAUUCCACCCTT
Negative control (siCtrl)	UUCUCCGAACGUGUCACGUTT	ACGUGACACGUUCGGAGAATT

### 2.7 Adenovirus construction and transfection

Recombinant adenoviruses containing MARCH5 gene (Ad‐MARCH5) and corresponding control (Ad‐Ctrl) used in this study were designed and produced by Vigene Biology. Transfection of adenoviral particles was performed according to the manufacturer's protocol.

### 2.8 Real‐time polymerase chain reaction (RT‐PCR) analysis

The total RNA was extracted from tissues or cell lysates using TRIzol (Invitrogen). One microgram of extracted RNA was reverse transcribed into cDNA using the PrimeScript RT Reagent Kit (Takara). The RT‐PCR assay was performed on the CFX96TM Real‐time PCR Detection System (Bio‐Rad) using EvaGreen Supermix Kit (Bio‐Rad). The specific primers were synthesized by Sangon and details were shown in Table 2. All samples were run in triplicate, and data were averaged. Relative mRNA expression was normalized to β‐actin.

**TABLE 2:** Primers for RT‐PCR

Genes	Forward sequence (5'‐3')	Reverse sequence (5'‐3')
r‐MARCH5	TAAGTGGGTTCACCAGGCTT	ATGGCCTACAACCTGCATCA
r‐β‐actin	ACTATCGGCAATGAGCGGTTC	ATGCCACAGGATTCCATACCC
h‐HIF‐1α	GAACGTCGAAAAGAAAAGTCTCG	CCTTATCAAGATGCGAACTCACA
h‐MARCH5	GTCCAGTGGTTTACGTCTTGG	CCGACCATTATTCCTGCTGC
h‐β‐actin	CATGTACGTTGCTATCCAGGC	CTCCTTAATGTCACGCACGAT

Abbreviations: h, human; r, rat.

### 2.9 Western blotting analysis

Cells were lysed by RIPA buffer (Beyotime) with protease and phosphatase inhibitor cocktail. Equal amounts of protein were loaded on 10% SDS‐PAGE gels and then transferred to PVDF membranes (Millipore). The membranes were incubated with corresponding primary antibodies and HRP‐conjugated secondary antibodies. Bands were detected by an ECL system and blotting density was determined using Bio‐Rad analysis software Image Lab.

### 2.10 Cell proliferation assay

Cell proliferation was detected using the enhanced cell counting kit‐8 (CCK8) assay (Beyotime) following the manufacturer's instructions. Cells were seeded in 96‐well plates at a density of 2 × 10^4^ per well. Transfection and treatment of cells were performed as described above. Then, CCK8 reagent was added to each well for a further 2 hours incubation. Subsequently, cell viability was evaluated by detecting the absorbance at 450 nm and quantified by calculating the absorbance percentage of the treated group vs control group.

### 2.11 Cell migration assay

The migration of ECs was evaluated using a wound healing assay. For migration, cells (5 × 10^5^) were seeded in 6‐well plates and grown to confluency. HCMECs monolayers were scratched with a sterile pipette tip. The remaining area of the scratch was measured using Image J software.

### 2.12 Tube formation assay

Matrigel (Corning) was pre‐coated and loaded into the 96‐well plates. After the gels solidified, cells (3 × 10^4^/well) treated with indicated conditions were plated on. The cells and intercellular junctions were observed and photographed using an inverted microscope. The tube length and other parameter were calculated by Image J with the Angiogenesis Analyzer plugin to analyse the tube formation.

### 2.13 Measurement of NO production

Intracellular NO production was detected using a total nitric oxide assay kit (Beyotime) according to the manufacturer's recommendations.

### 2.14 Statistical analysis

All data were presented as mean ± SD. Statistical analysis was performed using GraphPad Prism 7.0. Comparisons between two groups were analysed by Student's *t* test, and multigroup comparisons were analysed by one‐way ANOVA followed by the Dunnett's test. A value of *P* < .05 was considered statistically significant.

## 3 RESULTS

### 3.1 MARCH5 expression is decreased in heart tissues of MI rats and hypoxic endothelial cells

**FIGURE 1:**
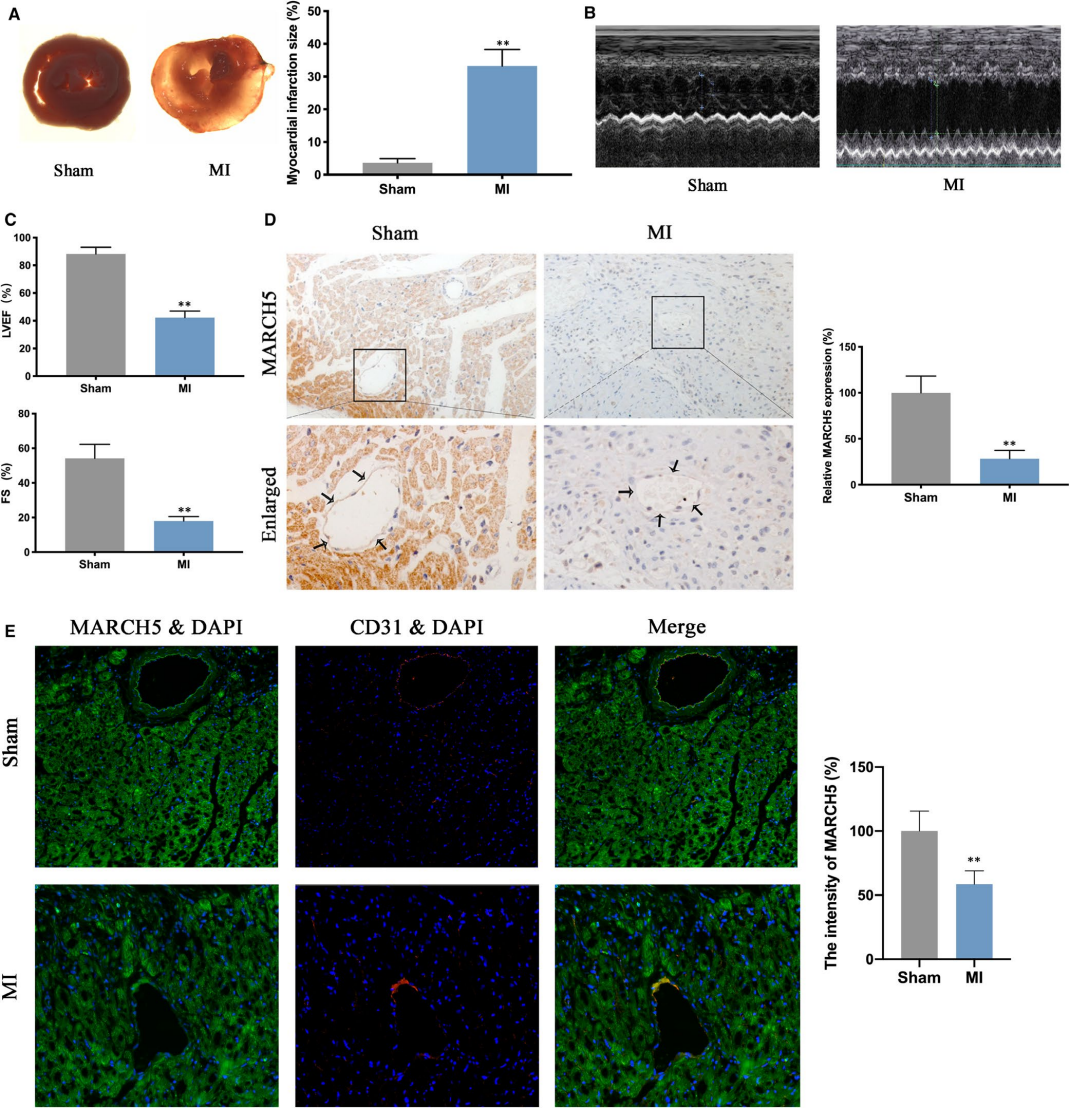
MARCH5 expression was decreased in rat myocardial infarction (MI) model. A, Myocardial infarct size was determined by TTC staining. B‐C, Cardiac function was determined by echocardiogram after 7 d left anterior descending coronary artery ligation in each group. D, Quantitative analysis of MARCH5 expression by immunohistochemistry (MARCH5‐brown, Nucleus‐blue). E, Co‐staining of MARCH5 and CD31, a classic endothelial cell marker (CD31‐red, MARCH5‐green, DAPI‐blue). Data were shown as mean ± SD (n ≥ 3), *P < .05, **P < .01 vs sham group

Firstly, TTC staining showed that rats with LAD coronary artery ligation exhibited increased infarct size (Figure 1A). In addition, the MI rats showed a lower LVEF and decreased FS compared with the sham group (Figure 1B,C). These results suggested the MI model was established successfully. Then, RT‐PCR and Western blot were used to quantify MARCH5 alterations in the context of ischaemia injury. Compared with the sham group, MARCH5 was significantly decreased in the infarct cardiac tissues both at transcription and protein expression levels (Figure S1A,B). Immunohistochemistry staining reconfirmed that MARCH5 expression was down‐regulated in response to the ischaemia injury (Figure 1D). To observe MARCH5 expression changes in cardiac ECs, CD31 and MARCH5 co‐staining was performed. As shown in Figure 1E, ischaemia injury significantly reduced MARCH5 expression in ECs.

**FIGURE 2:**
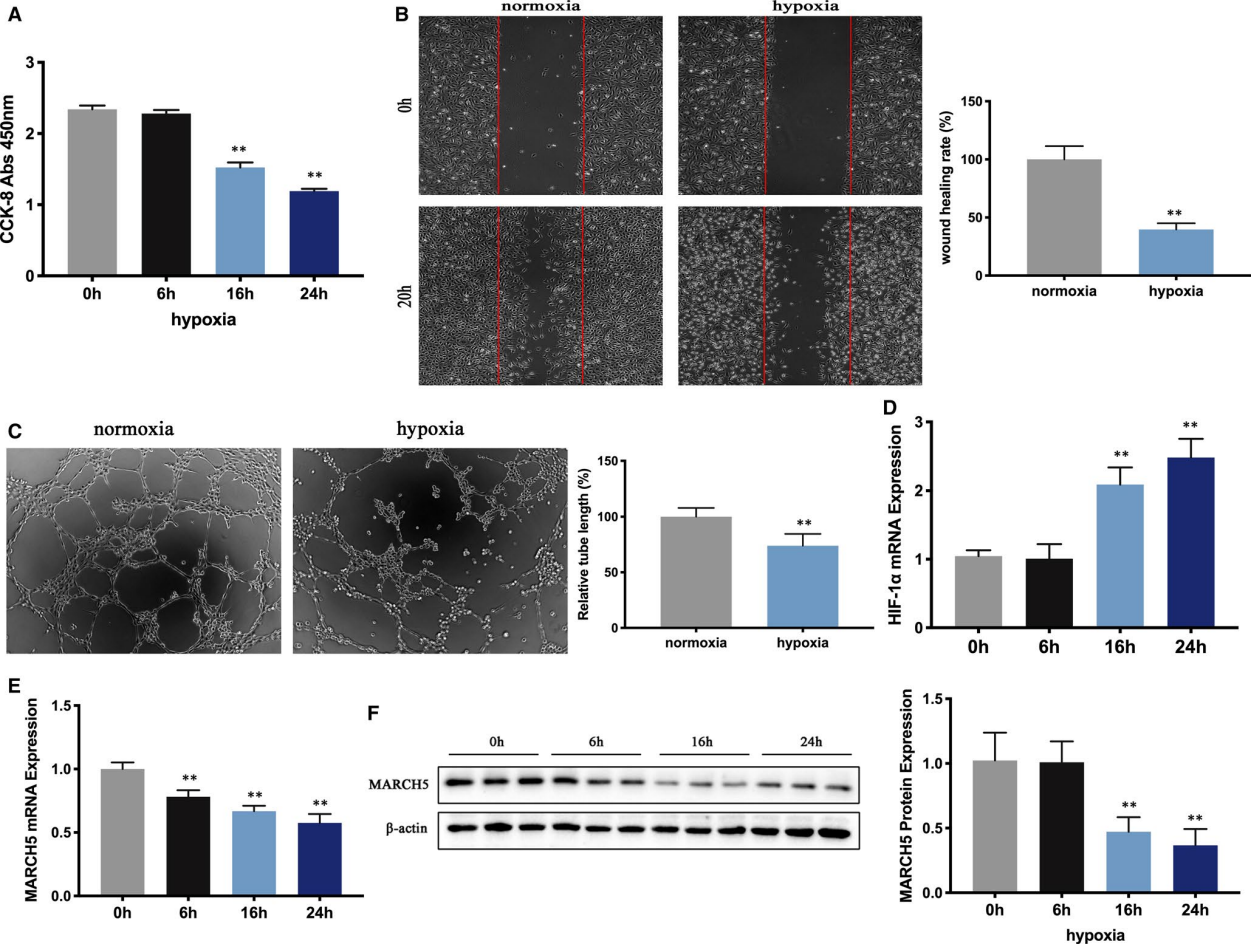
Hypoxia exposure leads to a time‐dependent down‐regulation of MARCH5 in human cardiac microcirculation endothelial cells (HCMECs). HCMECs were exposed to 1% O_2_ in groups for 0, 6, 16 and 24 h. A, Cell counting kit‐8 (CCK8) assay was performed to assess the cell viability. B, Wound healing assay under normoxia (20% O_2_) or hypoxia (1% O_2_) conditions for 20 h. C, Representative images showing the reduced tube formation of HCMECs under normal or hypoxia conditions. D, Increased expression of HIF‐1α mRNA in HCMECs under hypoxia conditions. E, Decreased expression of MARCH5 mRNA under hypoxic conditions. F, Decreased protein expression of MARCH5 under hypoxic conditions. Data were shown as mean ± SD (n ≥ 3), *P < .05, **P < .01 vs normoxia (hypoxia 0 h) group

To further investigate the role of MARCH5 in ischaemia injury, HCMECs were incubated with 1% O_2_ for 0, 6, 16 and 24 hours. From CCK8 assay, we observed that hypoxia reduced ECs proliferation by a time‐dependent way (Figure 2A). Meanwhile, the migration rates and tube formation ability of HCMECs were also decreased in hypoxia environment (Figure 2B,C). Moreover, hypoxia‐inducible factor 1α (HIF‐1α), a marker of hypoxia, revealed greater expression in hypoxia‐stimulated HCMECs (Figure 2D). These results indicated that HCMECs’ proliferation, migration and tube formation ability were seriously affected under hypoxia stress, the hypoxia model in vitro was established successfully. MARCH5 expression was further evaluated by RT‐PCR and Western Blot, as shown in Figure 2E,F, the expression of MARCH5 decreased gradually with the prolongation of hypoxia. These results provided us with a potential link between MARCH5 reduction and hypoxia injury.

### 3.2 Knockdown of MARCH5 deteriorates endothelial function

**FIGURE 3:**
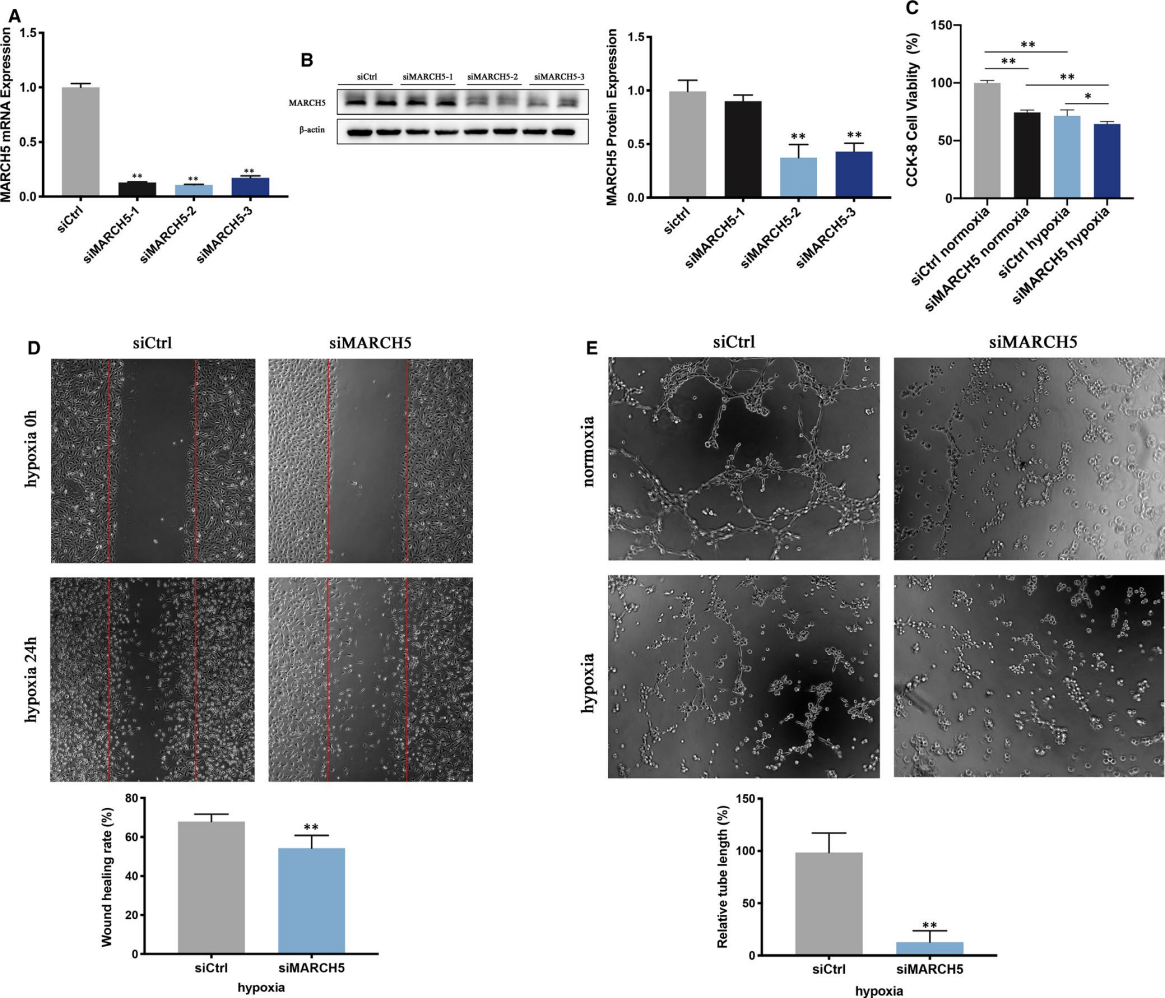
MARCH5 knockdown results in decreased proliferation, migration and tube formation of HCMECs. HCMECs were transfected with scrambled siRNA, MARCH5 siRNA1, MARCH5 siRNA2, MARCH5 siRNA3 for 48 h. A, RT‐PCR was performed to detect MARCH5 levels. B, Western blot analysis for MARCH5 expression. SiMARCH5‐2 was used for further studies. C, CCK8 assay for proliferation in ECs under normoxia and hypoxia conditions. D, Wound healing analysis for migration in ECs under hypoxia. E, Tube formation in ECs under normoxia or hypoxia. siCtrl, siMARCH5‐1, siMARCH5‐2 and siMARCH5‐3 labels indicate the scrambled siRNA group and MARCH5 siRNA groups, respectively. Data were shown as mean ± SD (n ≥ 3), *P < .05, **P < .01 vs siCtrl

To examine the effects of MARCH5 on ECs function in response to hypoxia, ECs were transfected with specific siRNAs of MARCH5 (siMARCH5) for 24 hours before hypoxia treatment. After transfection, MARCH5 expression was down‐regulated in siMARCH5 group compared with control (siCtrl) group, indicating that the transfection was successful (Figure 3A,B). The siMARCH5‐2 with the best silencing efficacy was selected for further studies. The ECs proliferation was inhibited in siMARCH5 group under both normoxia and hypoxia conditions compared with siCtrl group (Figure 3C). Then, ECs migration analysis using wound healing assay was performed, results revealed that the cell migration was reduced in siMARCH5 group (Figure 3D). Besides, we employed the EC tube formation assay on Matrigel matrix and found that MARCH5‐deficient ECs exhibited markedly blunted angiogenesis (Figure 3E). These results indicated that knockdown of MARCH5 impaired endothelial proliferation, migration and angiogenesis.

### 3.3 Overexpression of MARCH5 alleviates endothelial dysfunction

**FIGURE 4:**
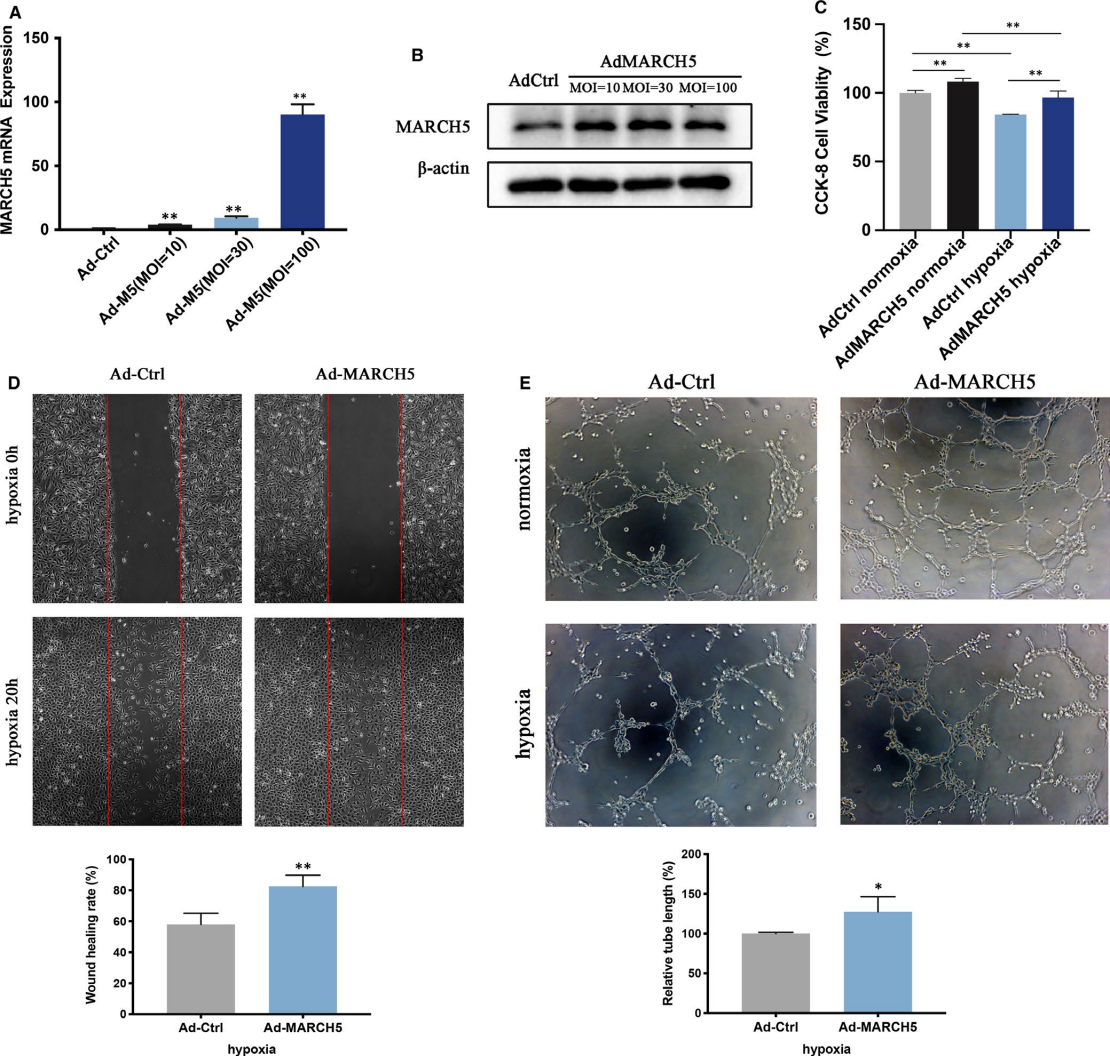
MARCH5 overexpression promotes proliferation, migration and tube formation of HCMECs. HCMECs were infected with Ad‐Control (Ad‐Ctrl) or Ad‐MARCH5 at different multiplicity of infection (MOI). A, RT‐PCR was performed to detect MARCH5 levels. B, Western blot analysis for MARCH5 expression, MOI = 10 was used for further studies. C, CCK8 assay for proliferation in ECs under normoxia and hypoxia conditions. D, Wound healing analysis for migration in ECs under hypoxia. E, Tube formation in ECs under normoxia or hypoxia. Data were shown as mean ± SD (n ≥ 3), *P < .05, **P < .01 vs Ad‐Ctrl

Because loss of MARCH5 mediated endothelial dysfunction, we wondered whether overexpression of MARCH5 could promote ECs function. Adenovirus encoding MARCH5 cDNA (Ad‐MARCH5) was transduced into ECs. Compared to control (Ad‐Ctrl) group, the protein and mRNA expression increased dramatically at all three different MOI (10, 30 and 100) in Ad‐MARCH5 group (Figure 4A,B). And the MOI of 10 was chosen as the optimal dose for subsequent studies. Likewise, CCK8, wound healing and tube formation assays were performed under hypoxia condition (Figure 4C‐E). ECs with MARCH5 overexpression displayed elevated proliferation, migration and angiogenesis compared to the vehicle, suggesting overexpression of MARCH5 could alleviate endothelial injury induced by hypoxia.

### 3.4 MARCH5 affects ECs function through eNOS‐dependent mechanism

**FIGURE 5:**
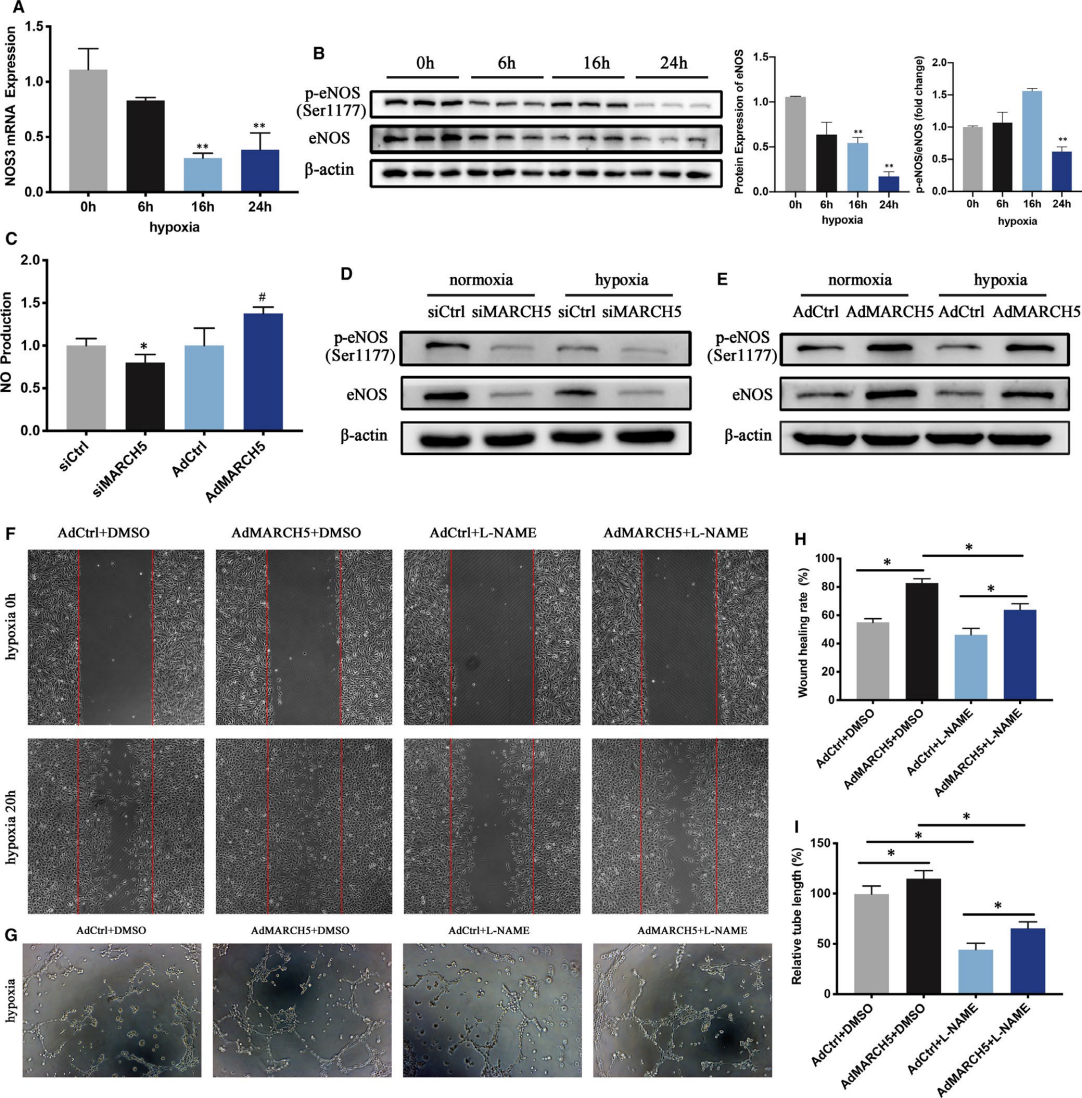
MARCH5 affects expression of eNOS in HCMECs under normoxia and hypoxia. A, Expression levels of NOS3 (eNOS) mRNA in ECs stimulated with indicated time was detected by RT‐PCR. B, Protein expression levels of eNOS and p‐eNOS were detected by Western blot under hypoxia. C, NO content was detected. D, Western blot demonstrated that knockdown of MARCH5 markedly decreased eNOS and p‐eNOS expression under both normoxia and hypoxia. E, Overexpression of MARCH5 significantly increased eNOS and p‐eNOS expression under both normoxia and hypoxia by Western blots. F, Wound healing assay was performed in MARCH5‐overexpression ECs under hypoxia in the presence or absence of L‐NAME (100 μmol/L). G, MARCH5‐overexpression ECs tube formation under hypoxia in the presence or absence of L‐NAME (100 μmol/L). H, Quantification of F. I, Quantification of G. NOS inhibitor L‐NAME aborted cellular proliferation and tube formation of Ad‐MARCH5 ECs vs Ad‐Ctrl group under hypoxia stress. Data were shown as mean ± SD (n ≥ 3), *P < .05, **P < .01 vs hypoxia 0 h or siCtrl, ^#^ *P* < .05 vs Ad‐Ctrl

Endothelial nitric oxide synthase (eNOS, also named nitric oxide synthase‐3 [NOS3]), one of the critical enzymes regulating nitric oxide (NO) production, plays a key role in ECs function.^23^, ^24^ Therefore, eNOS expression levels during hypoxia were measured next step. RT‐PCR showed eNOS mRNA was decreased during hypoxia (Figure 5A). The decrease in the expression of total eNOS and p‐eNOS at Ser1177 was also confirmed by western blot analysis (Figure 5B).

To investigate whether MARCH5 affects ECs function through eNOS signalling pathway, firstly, we explored the relationship between NO content and MARCH5 expression. Results showed that NO content in ECs was significantly decreased after silencing MARCH5 and significantly increased after transfection with Ad‐MARCH5 (Figure 5C). Secondly, eNOS expression levels after specific silencing or overexpression of MARCH5 were detected. Knockdown of MARCH5 attenuated the levels of eNOS, while overexpression MARCH5 increased the expression of eNOS in both normoxia and hypoxia conditions (Figure 5D,E). These data suggested that MARCH5 may affect ECs function through NO synthesis regulated by eNOS.

Furthermore, ECs were pre‐treated with NG‐nitro‐l‐arginine methyl ester (L‐NAME), an inhibitor of nitric oxide synthase (NOS), followed by hypoxia stimulation. Incubation with L‐NAME abolished MARCH5‐induced enhancement of migration and tube formation of ECs (Figure 5F‐I). These data indicated that MARCH5 regulates ECs function through the eNOS‐dependent mechanism.

### 3.5 MARCH5 regulates eNOS phosphorylation via Akt/eNOS pathway

**FIGURE 6:**
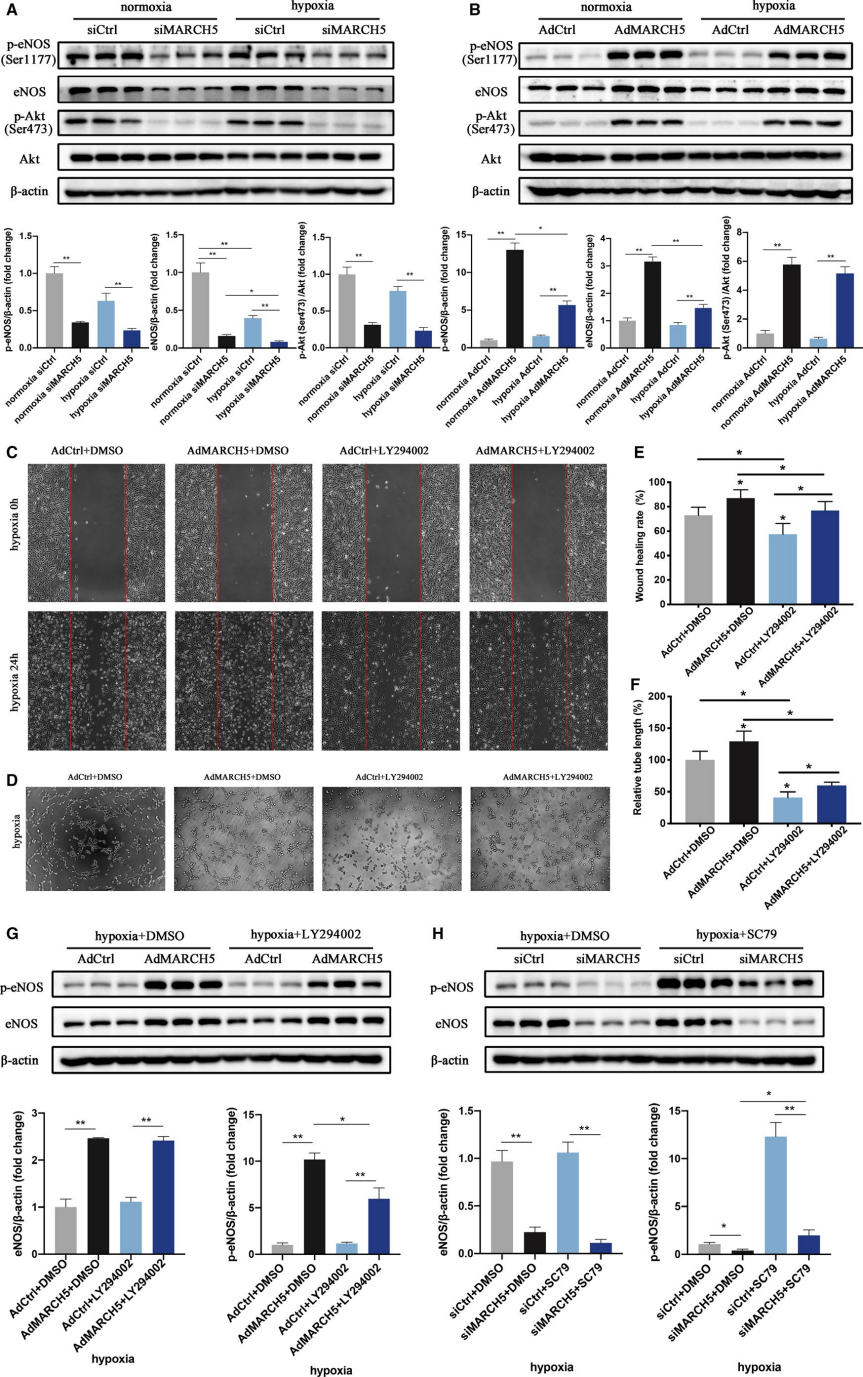
MARCH5 protects ECs against hypoxia injury via Akt/eNOS pathway. A, Knockdown of MARCH5 decreased eNOS, p‐eNOS and p‐Akt. B, Overexpression of MARCH5 up‐regulated eNOS, p‐eNOS and p‐Akt. Ad‐Ctrl and Ad‐MARCH5 ECs under hypoxia were incubated with DMSO or the Akt inhibitor LY294002 (10 μg/mL). C, Wound healing assay was performed in each group. D, Tube formation. E, F, Quantitative analysis of C and D. Akt phosphorylation inhibition with LY294002 aborted cellular proliferation and tube formation of Ad‐MARCH5 ECs vs Ad‐Ctrl group under hypoxia stress. G, Protein expression of eNOS and p‐eNOS in Ad‐Ctrl and Ad‐MARCH5 ECs incubated with DMSO or LY294002 under hypoxia. E, Protein expression of eNOS and p‐eNOS in siCtrl and siMARCH5 ECs incubated with DMSO or Akt activator SC79 (8 μmol/L) under hypoxia. Data were shown as mean ± SD (n ≥ 3), *P < .05, **P < .01

Akt is one of the most well‐known kinases, which phosphates and actives eNOS, whether MARCH5 affects eNOS through Akt/eNOS pathway is identified. And the effects of MARCH5 on Akt phosphorylation were evaluated by Western blot. Consistent with changes of p‐eNOS levels, knocking down of MARCH5 decreased the expression p‐Akt, while overexpression of MARCH5 increased the expression of p‐Akt. (Figure 6A,B). These results demonstrated that the protective role of MARCH5 under ischaemia/hypoxia injury may be ascribed to the regulation of Akt/eNOS signalling.

To further test our hypothesis, we treated ECs with an Akt phosphorylation inhibitor (LY294002) and an Akt phosphorylation agonist (SC79). As shown in Figure 6C‐F, administration with LY294002 damaged ECs migration and angiogenesis, while overexpression of MARCH5 can partially reverse the ECs injury caused by LY294002, suggesting that MARCH5 affected ECs function by regulating the phosphorylation of Akt. Furthermore, overexpression of MARCH5 increased p‐eNOS expression and this effect can be partly blocked by LY294002; similarly, knockdown of MARCH5 decreased eNOS phosphorylation and this effect can be partially reversed by Akt phosphorylation agonist SC79, suggesting MARCH5 protected ECs via Akt/eNOS pathway (Figure 6G,H).

## 4 DISCUSSION

**FIGURE 7:**
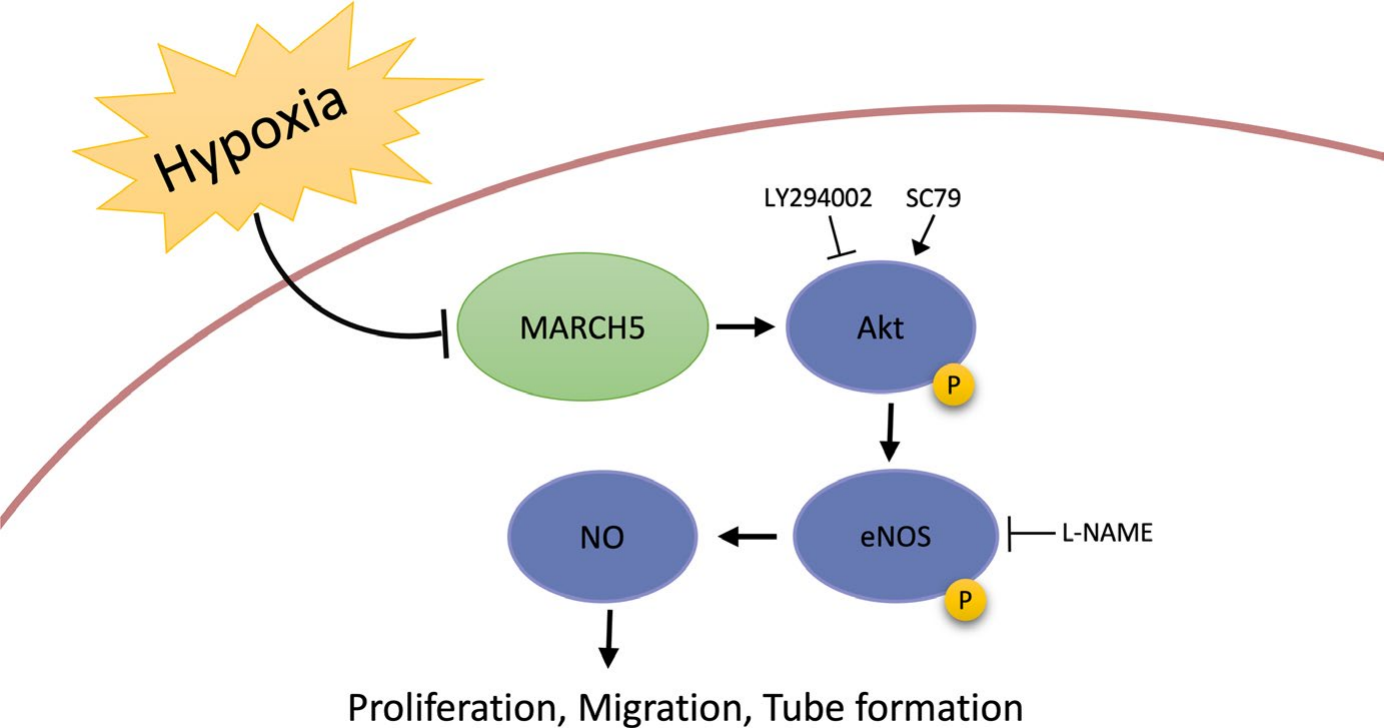
Schematic representation of the role of MARCH5 in ECs function. MARCH5 expression was decreased in ischemic and hypoxic ECs. Inhibition of MARCH5 expression aggravates ECs damage caused by hypoxia. Overexpression of MARCH5 ameliorates the injury induced by hypoxia. MARCH5 promotes NO production and protects endothelial cells by up‐regulating Akt/eNOS pathway

In the present study, the role of MARCH5 on ECs under ischaemia/hypoxia injury was investigated. We found the followings: (a) the expression of MARCH5 was decreased in rats with MI and ECs under hypoxia injury; (b) knockdown of MARCH5 aggravates ECs damage caused by hypoxia, overexpression MARCH5 ameliorates the injury induced by hypoxia, suggesting that MARCH5 plays a protective role in ECs function during hypoxia; (c) MARCH5 promotes NO production and protects endothelial cells by up‐regulating Akt/eNOS pathway. The main findings were illustrated in Figure 7.

MARCH5 is an important E3 ubiquitin ligase, apart from involvement in mitochondria fusion and fission, MARCH5 participated in regulating apoptosis, mitophagy and endoplasmic reticulum (ER) stress.^10^, ^11^, ^12^, ^25^ Studies showed that MARCH5 exerted potential regulator effects in hypoxia stimulation^15^, ^16^; however, the functions of MARCH5 in hypoxic ECs remain unknown. In our study, MARCH5 expression in ECs was markedly decreased under hypoxic stress. Simultaneously, our experiments demonstrated that MARCH5 expression was directly linked with ECs function, since knockdown of MARCH5 decreased cell proliferation, migration, tube formation and NO production, whilst overexpression of MARCH5 increased these parameters. These findings are partly consistent with previous studies, which reported that MARCH5 promoted the migration and invasion in ovarian cancer and breast cancer both in vitro and in vivo.^26^, ^27^ Park et al^14^ showed that MARCH5 contributes to cell survival under mitochondria stress conditions in HeLa cells. Recent studies demonstrated that MARCH5 also played a critical protectively role in cardiomyocytes under oxidative stress and ischaemia/reperfusion (I/R) injury,^13^, ^28^ whether the effects of MARCH5 on endothelial function was mediated partially or mainly through dysfunction of cardiomyocytes need a further investigation.

Another major finding in the present study is that Akt/eNOS pathway contributes to the development of MARCH5‐related ECs function changes. eNOS, one of the isoforms from nitric oxide syntheses (NOSs), plays a pivotal role in the maintenance of cardiovascular homeostasis through converting l‐arginine and oxygen into l‐citrulline and NO.^29^, ^30^ eNOS‐deficient^(−/−)^ mice showed significantly larger infarct size and more neutrophil accumulation in myocardial ischaemia‐reperfusion injury,^31^ overexpression of eNOS attenuated myocardial reperfusion injury,^32^ implying increasing eNOS pathway activation could constitute a promising strategy in MI. There are at least three mechanisms to regulate eNOS bioavailability, such as transcriptional regulation of eNOS, posttranscriptional activation of eNOS and reduction of ROS‐mediated breakdown of NO.^33^ In the present study, we demonstrated MARCH5 not only regulated NOS3 mRNA, eNOS protein expression but also eNOS phosphorylation modification (Figure S1C, Figure 6B). Given that phosphorylation of Ser1177 within eNOS is critical for activation of eNOS, efforts in understanding how MARCH5 might regulate p‐eNOS were made. The PI3K/Akt pathway mediated several cellular functions especially cell viability, proliferation and angiogenesis.^34^ Simultaneously, PI3K/Akt signalling is widely recognized to phosphorylate and activate eNOS,^23^, ^35^ leading to augmented NO synthesis and enhanced vasodilatation. PI3K/Akt signalling also participated in hypoxia/reoxygenation injury through improving eNOS‐mediated endothelial vasodilatation function.^36^ As shown in the results, we found that MARCH5 influenced the expression of phosphorylated Akt at both Ser473 and Thr308 residues (Figure 6A,B, Figure S1D,E). The relationship among MARCH5, p‐Akt and p‐eNOS was further explored. Inhibition of the Akt phosphorylation by LY294002 aborted the MARCH5 overexpression‐induced protective effects on ECs under hypoxia stress. Incubation with SC79, an Akt activator, significantly reversed the decreased p‐eNOS expression in the MARCH5‐knockdown group vs control. These data indicated that the expression of p‐eNOS modulated by MARCH5 was via Akt/eNOS pathway, at least in part.

Taken together, our study demonstrated the protective effect of MARCH5 on the endothelial cell function under hypoxia and MARCH5 played an important role in the regulation of eNOS partly through the Akt/eNOS pathway. To the best of our knowledge, this is an earlier study to reveal the correlation between MARCH5 and eNOS.

However, there are still many limitations. Firstly, our experiments were mainly carried out in vitro, it couldn't fully mimic the ischemic environment in vivo, animal studies should be performed for further investigation. Secondly, NO generation was detected indirectly by assessing NO2‐/NO3‐ levels in cellular assays. More sensitive assays are certainly needed in subsequent studies on NO homeostasis. Thirdly, considering the complex regulatory network of eukaryotes, we couldn't rule out the possibility that other molecular mechanisms may also be involved in, further studies are necessary.

## 5 CONCLUSIONS

This study firstly proved that MARCH5 played a protective role in maintaining ECs function under hypoxic stress by regulating Akt/eNOS signalling, which might be a potential therapeutic target for the clinical management of ischemic heart diseases.

## CONFLICT OF INTEREST

The authors declare that there are no conflicts of interest.

## AUTHOR CONTRIBUTIONS

**Wenhua Lei:** Conceptualization (lead); Investigation (lead); Methodology (lead); Writing‐original draft (lead). **Junli Li:** Supervision (equal); Writing‐review & editing (equal). **Chang‐Ming Li:** Conceptualization (equal); Formal analysis (equal); Funding acquisition (equal). **Li Chen:** Formal analysis (equal). **Fang‐Yang Huang:** Formal analysis (equal); Funding acquisition (equal). **Dan Xiao:** Formal analysis (equal); Resources (equal). **Jialiang Zhang:** Formal analysis (equal); Writing‐original draft (supporting). **Jiahao Zhao:** Formal analysis (supporting). **Guoyong Li:** Formal analysis (supporting). **Tianyi Qu:** Methodology (equal). **Hao Zhou:** Methodology (equal). **Yanbiao Liao:** Funding acquisition (equal); Writing‐review & editing (equal). **Mao Chen:** Conceptualization (equal); Writing‐review & editing (equal).

## Supporting information

Fig S1

## Data Availability

The data that support the findings of this study are available from the correspondence author on reasonable request.
