# Supplementary material for: MARCH5 restores endothelial cell function against ischaemic/hypoxia injury via Akt/eNOS pathway
Source: J Cell Mol Med. 2021 Feb 21;25(7):3182–93. doi: 10.1111/jcmm.16386 (PMC8034466; doi:10.1111/jcmm.16386)
Supplement: Supplementary file 1 — Fig S1 [file JCMM-25-3182-s001.docx]

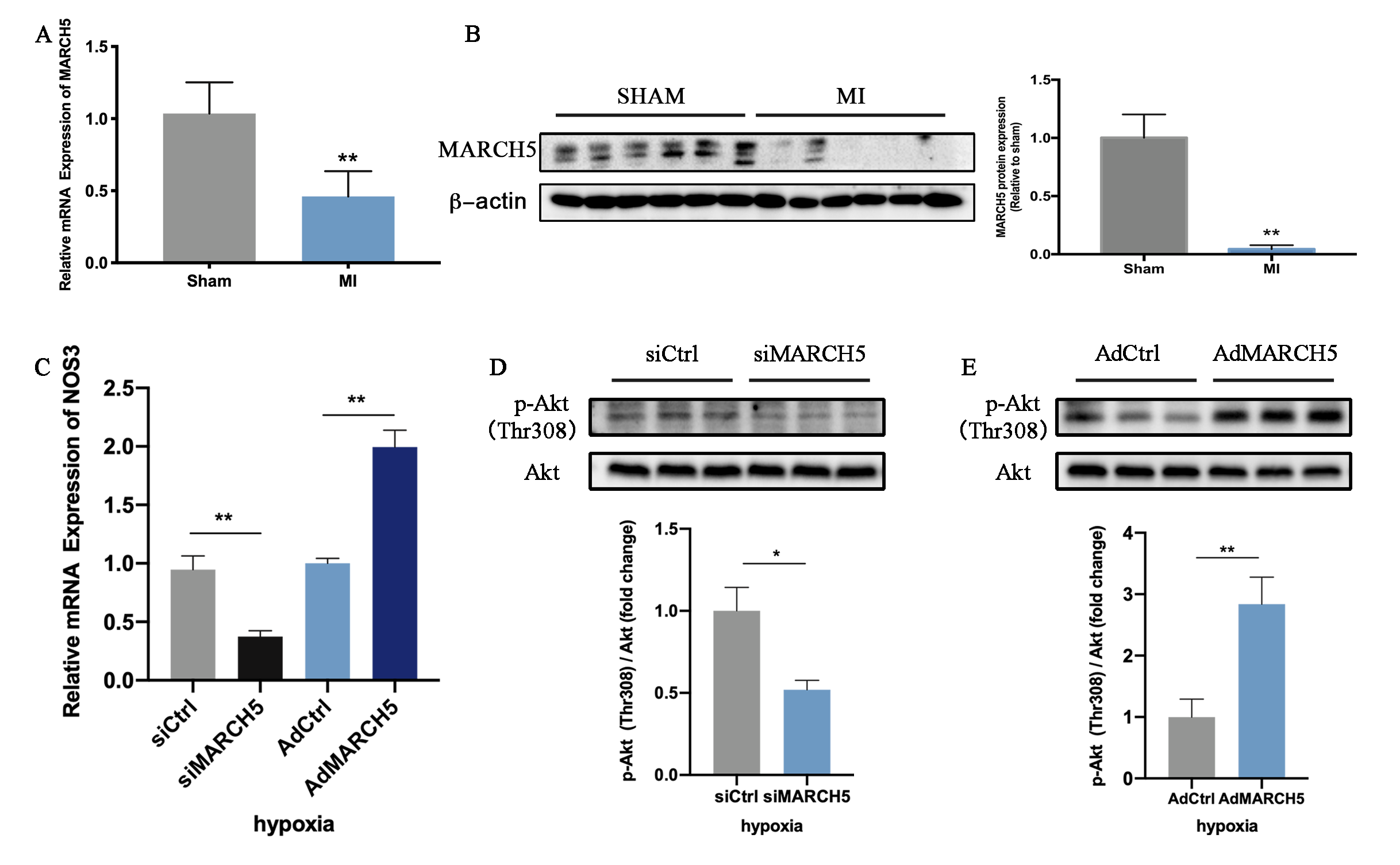


**Supplementary Figure S1.** (A) MARCH5 mRNA level was determined by RT-PCR in sham and MI group. (B) MARCH5 protein expression was detected by Western blot. The expression of MARCH5 was markedly decreased in the MI group compared with the sham group. (C) Quantitation of NOS3 (eNOS) mRNA in different groups under hypoxic condition by RT-PCR. (D) Knockdown of MARCH5 decreased p-Akt (Thr308) in ECs under hypoxia stress. (E) Overexpression of MARCH5 up-regulated p-Akt (Thr308) in ECs under hypoxia. Data were shown as mean ± SD (n≥3), **P*<0.05, ***P*<0.01
